# Color stability of ceramic brackets immersed in potentially staining solutions

**DOI:** 10.1590/2176-9451.20.4.032-038.oar

**Published:** 2015

**Authors:** Bruna Coser Guignone, Ludimila Karsbergen Silva, Rodrigo Villamarim Soares, Emilio Akaki, Marcelo Coelho Goiato, Matheus Melo Pithon, Dauro Douglas Oliveira

**Affiliations:** 1 MSc in Orthodontics, Pontifícia Universidade Católica de Minas Gerais (PUC Minas), Belo Horizonte, Minas Gerais, Brazil; 2 Associate professor, Pontifícia Universidade Católica de Minas Gerais (PUC Minas), Belo Horizonte, Minas Gerais, Brazil; 3 Full professor, Universidade Estadual Paulista Júlio de Mesquita Filho (UNESP), Araçatuba, São Paulo, Brazil; 4 Adjunct professor of Orthodontics, Universidade Estadual do Sudoeste da Bahia (UESB), Jequié, Bahia, Brazil; 5 Director of Orthodontics Program, Pontifícia Universidade Católica de Minas Gerais (PUC Minas), Belo Horizonte, Minas Gerais, Brazil

**Keywords:** Orthodontic brackets, Optical properties, Color instability

## Abstract

**OBJECTIVE:**

To assess the color stability of five types of ceramic brackets after immersion in potentially staining solutions.

**METHODS:**

Ninety brackets were divided into 5 groups (n = 18) according to brackets commercial brands and the solutions in which they were immersed (coffee, red wine, coke and artificial saliva). The brackets assessed were Transcend (3M/Unitek, Monrovia, CA, USA), Radiance (American Orthodontics, Sheboygan, WI, USA), Mystique (GAC International Inc., Bohemia, NY, USA) and Luxi II (Rocky Mountain Orthodontics, Denver, CO, USA). Chromatic changes were analyzed with the aid of a reflectance spectrophotometer and by visual inspection at five specific time intervals. Assessment periods were as received from the manufacturer (T_0_), 24 hours (T_1_), 72 hours (T_2_), as well as 7 days (T_3_) and 14 days (T_4_) of immersion in the aforementioned solutions. Results were submitted to statistical analysis with ANOVA and Bonferroni correction, as well as to a multivariate profile analysis for independent and paired samples with significance level set at 5%.

**RESULTS:**

The duration of the immersion period influenced color alteration of all tested brackets, even though these changes could not always be visually observed. Different behaviors were observed for each immersion solution; however, brackets immersed in one solution progressed similarly despite minor variations.

**CONCLUSIONS:**

Staining became more intense over time and all brackets underwent color alterations when immersed in the aforementioned solutions.

## Abstract (pt)

**OBJETIVO:**

avaliar a estabilidade da cor de cinco tipos de braquetes cerâmicos após imersão em soluções potencialmente corantes.

**MÉTODOS:**

noventa braquetes foram divididos em 5 grupos (n = 18) de acordo com a marca comercial dos braquetes e as soluções em que foram imersos (café, vinho tinto, Coca-Cola e saliva artificial). Os baquetes avaliados foram Transcend (3M / Unitek, Monrovia, CA, EUA), Radiance (American Ortodontia, Sheboygan, WI, EUA), Mystique (GAC International Inc., Bohemia, NY, EUA) e Luxi II (Rocky Mountain Orthodontics, Denver, CO, EUA). Alterações cromáticas foram analisadas com o uso de um espectrofotômetro de reflectância e por inspecção visual, em cinco intervalos de tempo específicos. Os momentos de avaliação foram: 24 horas (T_1_), 72 horas (T_2_), 7 dias (T_3_) e 14 dias (T_4_) de imersão nas soluções. Os resultados foram submetidos à avaliação estatística com análise de variância e correção de Bonferroni, bem como a uma análise do perfil multivariada para amostras independentes e pareadas, com nível de significância de 5%.

**RESULTADOS:**

a duração do período de imersão influenciou na alteração de cor de todos os braquetes testados, mesmo admitindo-se que essas alterações nem sempre puderam ser observadas visualmente. Diferentes comportamentos foram observados em cada solução de imersão; no entanto, braquetes imersos em um mesmo tipo de solução progrediram de forma semelhante, apesar das pequenas variações.

**CONCLUSÕES:**

a coloração se tornou mais intensa com o tempo, e todos os braquetes sofreram alteração de cor nas soluções imersas.

## INTRODUCTION

The increasing number of adult patients seeking orthodontic treatment has reinforced the need for esthetic orthodontic appliances.^1^ The orthodontic industry, aiming to profit from this demand, has invested in the development of different types of esthetic material, striving to meet the expectations of patients and clinicians.^1^^,^ 2 Ceramic brackets are a result os this process.^3^^,^ 4

Ceramic brackets may be manufactured with polycrystalline ceramic or monocrystalline sapphire.^3^^,^ 4 ^,^ 5 These brackets are inert to the oral environment^1 ^and have become the most commonly used esthetic fixed orthodontic appliances worldwide due to superior esthetics and mechanical resistance when compared to plastic brackets.^1^^,^ 3 ^-^ 6

Despite remarkable quality improvement since the introduction of the first ceramic brackets in the 80's, ceramic brackets currently available on the market still present significant limitations, such as high friability; increased friction with orthodontic wires, when compared to metallic brackets; the possibility of causing wear on antagonist teeth; and the risk of damaging the enamel structure during debonding.^7^^,^ 8 ^,^ 9 Although their color features are their major advantage over metallic brackets, there is a limited number of reports analyzing their optical properties over time.^10^ Lack of such studies may be related to technical difficulties in measuring brackets color, given that their geometry may hinder accurate color measurement with a spectrophotometer or colorimeter.^12^

Therefore, the objective of this study was to assess the color stability of different ceramic brackets exposed to potentially staining solutions during a period of time.

## MATERIAL AND METHODS

Ninety maxillary central incisor ceramic brackets of five different types and commercial brands (Table 1) were assessed (n = 18). Prior to immersion in potentially staining solutions, all brackets had their bases leveled by 180-grain water sandpapers (Doble A^(r)^, Argentine Abrasives S.A.I.C, Argentina) in a polishing machine (Knuth-Rotor^(r)^, Struers, Denmark) under constant irrigation. Subsequently, all brackets were cleaned with acetone to remove any speck adhered to their surface.

**Table 1.** Ceramic brackets evaluated.

Commercial brand	Composition	Manufacturer
Clarity	Polycrystalline alumina, stainless steel slot	3M/Unitek, Monrovia, CA, USA
Transcend	Polycrystalline alumina	3M/Unitek, Monrovia, CA, USA
Radiance	Monocrystalline alumina	American Orthodontics, Sheboygan, WI, USA
Mystique	Polycrystalline alumina	GAC International Inc., Bohemia, NY, USA
Luxi II	Polycrystalline alumina, gold slot	Rocky Mountain Orthodontics, Denver, CO, USA

## Staining analysis

Brackets were immersed in four potentially staining solutions: red wine (Miolo Terranova 2006, Bento Gonçalves, Brazil), coffee (Café Três Corações, Três Corações, Brazil), coke (Coca-Cola, Belo Horizonte, Brazil) and artificial saliva (control group). Each solution was distributed into five small black plastic containers, so as to eliminate light interference. Each container had six brackets of the same brand and was stored at room temperature. Solutions were changed every 24 hours. All brackets were divided into four groups, according to the solution in which they were immersed (n = 6). 

Before immersion (T_0_), all brackets had color measured (baseline - T_0_). Subsequently, color was analyzed after a period of 24 (T_1_), and 72 hours (T_2_), as well as after 7 (T_3_) and 14 days (T_4_) of immersion.

Color readings were assessed with the aid of a reflectance spectrophotometer (UV-visible spectrophotometer UV-2450, Shimadzu, Kyoto, Japan), according to the Commission Internationale de l´Eclairage (CIE) L*, a*, b* (LAB) color scale.^15^ The CIELAB system of color assessment quantitatively assess the color features of an object, based on three parameters (L*, a* e b*): L* is the measurement of brightness quantified on a scale in which black has an L* value equal to zero, whereas a totally reflected light has an L* value equal to 100; a* measures the amount of red (+a*) and green (-a*); and b* measures the amount of yellow (+b*) and blue (-b*). Total color changes (Δ E*ab) were calculated by the following equation:^16^Δ E*ab = [(Δ L*)² + (Δ a*)² + (Δ b*)²]1/2.

Changes in color parameters (Δ L*, Δ a* and Δ b*) were calculated by subtraction (i.e. T_1_ - T_0_). Prior to each measurement carried out with the reflectance spectrophotometer, all brackets were washed with distilled water, so as to remove any staining solution residue from their surface.

## Visual assessment

Visual analysis of brackets was performed at the same time intervals previously described and by two different operators. One bracket from each group was washed with distilled water, air-dried and placed on a white surface beside a similar bracket, which had not been immersed in any staining solution, for comparison.

This analysis aimed to visually detect potential bracket staining and relate it to the time of immersion in different solutions. Whenever any visible color change was detected, it was recorded as described by Mancuso *et al*.^11^

## Statistical analysis

Data were analyzed by means of SPSS 15.0, Microsoft Excel and Gpower 3.0. Multivariate profile analysis, analysis of variance (ANOVA) and *t test* for independent paired samples, with significance level set at 5%, were used to compare intra and intergroup mean values obtained in the reflectance spectrophotometer after immersion of brackets in different solutions during specific time intervals.

ANOVA was used to investigate differences between groups and when significant differences were found, *Bonferroni* correction was used to verify in which group such differences had occurred (Table 2). Multivariate profile analysis (Table 3) was used to analyze time effect not considering brackets brand. It was also used to test whether the staining pattern and the tested brackets brands were similar or different over time.

**Table 2.** Mean values of color alteration of brackets immersed in different solutions and over different periods of time.

		ΔE_1_ (24h)	ΔE_2_ (72h)	ΔE_3_ (7d)	ΔE_4_ (14d)
Clarity	Saliva	126.93	126.93	126.93	126.93
	Coke	117.53	104.93	98.06	46.38
	Coffee	109.86	90.31	54.93	42.24
	Red wine	40.5	50.84	72.94	135.31
Luxi II	Saliva	142.3	142.3	142.3	142.3
	Coke	88.41	108.6	98.19	29.68
	Coffee	78.16	48.88	74.13	58.15
	Red wine	26.84	43.42	65.63	140.72
Mystique	Saliva	116.47	116.47	116.47	116.47
	Coke	120.81	135.18	129.67	36.67
	Coffee	78.19	67.65	59.67	37.43
	Red wine	58.61^b^	65.71	73.39	112.14
Radiance	Saliva	155.07^e^	155.07^e^	155.07^e^	155.07^e^
	Coke	173.45^be^	166.85^abe^	175.58^abce^	55.92
	Coffee	96.95	74.78	40.79	85.03
	Red wine	80.52^abe^	75.39^b^	65.96	97.34
Transcend	Saliva	111.65	111.65	111.65	111.65
	Coke	75.57	93.5	96.78	50.92
	Coffee	44.74	45.6	44.12	94.18^c^
	Red wine	40.56	67.13	61.98	126.09

ANOVA and Bonferroni correction. Statistically significant differences (p < 0.05) between bracket brands are represented by letters.

**Table 3.** Multivariate analysis of brackets exposed to different staining solutions: time and brand factors.

		Significance	
	Coke	Coffee	Wine
Time (initial)	0.000	0.004	0.000
24 hours - 72 hours	0.363	0.007	0.000
72 hours - 7 days	0.801	0.002	0.002
7 days - 14 days	0.000	0.650	0.000
Time by brand (parallelism)	0.020	0.002	0.004
24 hours - 72 hours	0.486	0.478	0.018
72 hours - 7 days	0.525	0.009	0.012
7 days - 14 days	0.002	0.014	0.005
Brands	0.000	0.424	0.354

Coke: 1) Mauchly's sphericity test (p = 0.152); 2) non-significant Levene's test [?E1 (24 h) (p = 0.03); ?E2 (72 h) (p = 0.08); ?E3 (7 d) (p = 0.22); ?E4 (14 d) (p = 0.79)]. Coffee: 1) Mauchly's sphericity test (p = 0.001); 2) non-significant Levene's test [?E1 (24 h) (p = 0.58); ?E2 (72 h) (p = 0.37); ?E3 (7 d) (p = 0.32);?E4 (14 d) (p = 0.98)]. Wine: 1) Mauchly's sphericity test (p < 0.001); 2) non-significant Levene's test [?E1 (24 h) (p = 0.02); ?E2 (72 h) (p = 0.82); ?E3 (7 d) (p = 0.97); ?E4 (14 d) (p = 0.18)].

## RESULTS

After being immersed in artificial saliva for 24 hours, Radiance brackets presented statistically significant color alteration when compared to Transcend brackets which were the most stable group (Table 2).

ANOVA results (Table 2) revealed that, when immersed in coke, Radiance brackets presented statistically significant (*p* < 0.05) color alterations in comparison to other bracket brands after 24 and 72 hours, as well as after 7 days of immersion. However, there were no statistically significant differences (*p* > 0.05) regarding color changes between brackets brands in this solution after 14 days (Table 2). Immersion in coffee only caused statistically significant color alterations in Transcend brackets compared to Mystique brackets after 14 days of immersion (Table 2). When immersed in red wine for 24 hours, Radiance brackets presented statistically higher (*p* < 0.05) color alterations in comparison to Luxi II. The same interval of red wine immersion promoted statistically higher (*p* < 0.05) color alterations in Radiance brackets in comparison to Clarity, Luxi II and Transcend brackets. However, after 72 hours, the color alterations observed in Radiance brackets were only higher than Luxi II brackets (Table 2), and differences between bracket brands after this period of immersion were not found.

Multivariate analysis results (Table 3) revealed that, during a specific time period (from 7 to 14 days),there were significant color changes (*p* = 0.000) in all brackets immersed in coke. Immersion in the same solution led to similar staining patterns after 24 to 72 hours (*p* = 0.486), and from 72 hours to 7 days (*p* = 0.525), although a different staining pattern occurred from 7 to 14 days (*p* = 0.002). A time effect (*p* = 0.004) was also observed on brackets exposed to coffee solution, since there were significant color alterations for specific time periods (24 to 72 hours - *p* = 0.007; 72 hours to 7 days - *p* = 0.002). Immersion in the same solution led to a similar staining pattern on bracket brands only from 24 to 72 hours (*p* = 0.478). Finally, immersion in red wine also revealed a time effect (*p* = 0.000), since significant color alterations at all time intervals (24 to 72 hours - *p* = 0.000; 72 hours to 7 days - *p* =0.002; 7 to 14 days - *p* = 0.000) were observed in the brackets tested. Exposure to this solution also led to different (*p* = 0.004) staining patterns on bracket brands at all time intervals (24 to 72 hours, *p* = 0.018; 72 hours to 7 days, *p* = 0.012; 7 to 14 days, *p* = 0.005).

## Visual inspection

**Figure 1.**
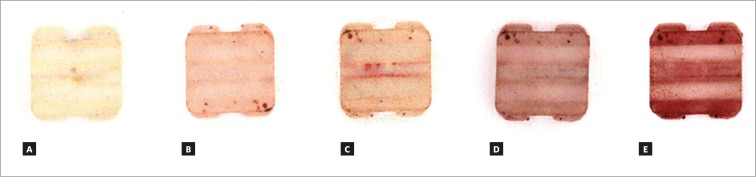
Brackets after immersion in red wine: A) baseline, B) after 24 hours, C) after 72 hours, D) after 7 days, and E) after 14 days of immersion.

After seven days of immersion in staining solutions, chromatic changes were found in all types of brackets analyzed. Thereafter, there was progressive staining of brackets until 14 days of immersion. Brackets immersed in artificial saliva revealed no visible color changes after 24 hours of immersion (Fig 1).

## DISCUSSION

Ceramic brackets are used in Orthodontics when patients require an esthetic alternative to metallic brackets. Although color stability throughout orthodontic treatment is a very important characteristic of this type of material,^12^ very little is found in the literature about this property. Therefore, the aim of the present study was to assess color stability of different ceramic brackets immersed in potentially staining solutions.

Assessment of orthodontic material color stability may be performed with equipment especially designed to analyze the reflective characteristics of an object or by means of comparative visual assessment.^12^ Reflectance spectrophotometer and colorimeter are usually used for color analysis because these instruments provide consistent numerical evaluation of color characteristics.^13^^,^ 14

Previous studies have tried to correlate numeric color alteration readings (ΔE*) provided by a spectrophotometer to the visual perception of staining of composite resin or prosthetic restorations.^15^^-^ 19 In visual perception, a threshold of color alteration reflected in esthetic restorations with a mean ΔE* value greater than 2.0 is noticed by all observers, while ΔE* values ranging between 1.0 and 2.0 are not often perceived.^18^ Nevertheless, other authors have proposed ΔE* values as from 3.3^20^ and 3.7^21^ for clinical perception of color changes involving composite resin restorations. Thus, research in the orthodontic field can use these references to assess color stability of esthetic brackets and elastics.

Faltermeier *et al* 22 investigated color stability of esthetic brackets after ultraviolet light irradiation and exposure to staining solutions.^13^ The authors considered ΔE* mean alues ≥ 3.3 as clinically unacceptable. However, Lee^23^ assessed color changes in reflected and transmitted color, in addition to color parameters of esthetic brackets after thermocycling, and suggested ΔE* equal to 3.7 was the threshold for clinical perception of color alteration.

Importantly, the ΔE* values used as reference in previous studies may not be compared to those used in the present study, since in addition to using different types of brackets, our spectrophotometric assessment was performed on bracket worn bases, while the others measured it on bracket buccal surfaces. Assessment on bracket buccal surface may be influenced by the shadows of the bracket slot and wings. These areas also present greater potential for accumulation of staining pigments, which may camouflage the actual staining of the ceramic brackets structure. We decided to level the bracket bases to increase the precision of spectrophotometer assessment, since this equipment was developed to analyze the characteristics of light reflected on flat surfaces.

In addition to assessment carried out with the aid of a spectrophotometer, two calibrated operators also performed a visual analysis. Staining of all ceramic brackets was observed in all three staining solutions after the seventh day of immersion. Thereafter, a progressive staining of these brackets was visually observed by the end of the 14-day period. Red wine was the solution that caused the most intense staining of all brackets tested, followed by coffee and coke, respectively. Despite presenting the lowest pH levels among the three staining solutions tested and potentially affecting a given material surface, coke did not cause as much color alteration as coffee and red wine, probably due to lack of yellow pigment in its composition.^22^^,^ 25

As previously mentioned, the literature on color stability of orthodontic material is limited.^22^^,^ 25 However, several techniques have been described to study the staining of dental material. Methods of aging acceleration, such as thermocycling, immersion in artificial saliva, coffee, tea, grape juice and chlorhexidine, have been used in *in vitro* simulations.^14^ It has been demonstrated that the type of solution as well as total exposure time influenced the degree of color alteration of these types of material.^26^ These results are in accordance with our findings.

Ertas et al^15^ assessed color stability of five types of composite resins immersed in tea, coke, coffee, red wine and water. Similarly to the present study, these solutions were used because they are the potentially staining solutions frequently consumed by adults. The authors also established 14 days as the total immersion time due to believing it would initially resemble the environmental color stability challenge that composite resins must face in the oral cavity.

Although Radiance brackets showed greater ΔE* mean values of color alteration when compared to the other brackets after 24 and 72 hours and 7 days of immersion in coke, this difference was not statistically significant after 14 days. Bracket brand staining in coffee solution was similar, since only one single statistically significant difference was detected (14 days, Transcend *versus* Mystique, *p* = 0.9418). After 24 hours of immersion in red wine, Mystique brackets presented with significant color alteration in comparison to Luxi II. After the same period of time, as well as after 72 hours and 7 days of immersion in this solution, Radiance brackets stood out statistically with a higher color alteration in comparison to other bracket brands (Table 2). It was also possible to observe that, in general, time significantly affected color alteration of these brackets, and the pattern of color change in specific solutions and time periods was similar or different (Table 3).

Regarding the staining potential of each solution, an interesting fact was observed. Coke, which was the solution that caused the least color alteration during visual inspection, yielded the highest ΔE* values in the spectrophotometric analysis. A possible explanation for this observation is that, due to its acidic properties, this solution has the ability of altering the material surface, leading to greater absorption of coloring pigments from the solution by the porcelain, which can be detected accurately by the spectrophotometer while not detected by the human eye. In agreement with previous studies, it was visually observed in the present study that red wine caused more color alterations than coffee, which was also confirmed by the spectrophotometric analysis.

It is important to point out that these results should not be extrapolated to clinical reality, given that methodological limitations are inevitable when assessing color alterations of brackets *in vitro*. Reproducibility of the conditions present in the oral cavity is quite complex due to several factors, including the intricate flora and its by-products, in addition to biofilm deposition in the tested material. Therefore, further clinical studies investigating orthodontic material color stability should be conducted in order to keep up with orthodontic patients' demand.

## CONCLUSION

Ceramic brackets displayed color changes after immersion in staining solutions, and the period of exposure to red wine influenced the amount of staining registered in the ceramic brackets assessed. When immersed in coke and red wine for specific periods of time, Radiance brackets generally presented statistically higher color alterations in comparison to other bracket brands.
